# Spatial distribution and size of small canopy gaps created by Japanese black bears: estimating gap size using dropped branch measurements

**DOI:** 10.1186/1472-6785-13-23

**Published:** 2013-06-10

**Authors:** Kazuaki Takahashi, Kaori Takahashi

**Affiliations:** 1 Faculty of Tourism and Environmental Studies, Nagano University, 658-1 Shimonogo, Ueda, Nagano 386-1298, Japan; 2 Division of Gene Research, Department of Life Science, Research Center for Human and Environmental Sciences, Shinshu University, 3-15-1 Tokida, Ueda, Nagano 386-8567, Japan

**Keywords:** Animal–plant interaction, Bear shelf, Canopy disturbance, Gap distribution, Gap formation, Gap height, Topography

## Abstract

**Background:**

Japanese black bears, a large-bodied omnivore, frequently create small gaps in the tree crown during fruit foraging. However, there are no previous reports of black bear-created canopy gaps. To characterize physical canopy disturbance by black bears, we examined a number of parameters, including the species of trees in which canopy gaps were created, gap size, the horizontal and vertical distribution of gaps, and the size of branches broken to create gaps. The size of black bear-created canopy gaps was estimated using data from branches that had been broken and dropped on the ground.

**Results:**

The disturbance regime was characterized by a highly biased distribution of small canopy gaps on ridges, a large total overall gap area, a wide range in gap height relative to canopy height, and diversity in gap size. Surprisingly, the annual rate of bear-created canopy gap formation reached 141.3 m^2^ ha^–1^ yr^–1^ on ridges, which were hot spots in terms of black bear activity. This rate was approximately 6.6 times that of tree-fall gap formation on ridges at this study site. Furthermore, this rate was approximately two to three times that of common tree-fall gap formation in Japanese forests, as reported in other studies.

**Conclusions:**

Our findings suggest that the ecological interaction between black bears and fruit-bearing trees may create a unique light regime, distinct from that created by tree falls, which increases the availability of light resources to plants below the canopy.

## Background

Tree-fall canopy gaps, which are openings in the canopy caused by the fall of one or more trees, play a major role in the maintenance of forest ecosystems and biodiversity (e.g. [1]). The formation of canopy gaps creates heterogeneity in the abiotic environment, increasing both the availability of soil moisture and nutrients (e.g. [2]), and the amount of light passing through the canopy (e.g. [3]). This mechanism also provides a wide range of regeneration sites for tree species that have different environmental requirements (e.g. [1]). The gap disturbance regime influences the germination of seeds buried in soil (e.g. [4]), the growth of seedlings and saplings (e.g. [5]), and the replacement and coexistence of competing tree species [1,6-8].

**Figure 1:**
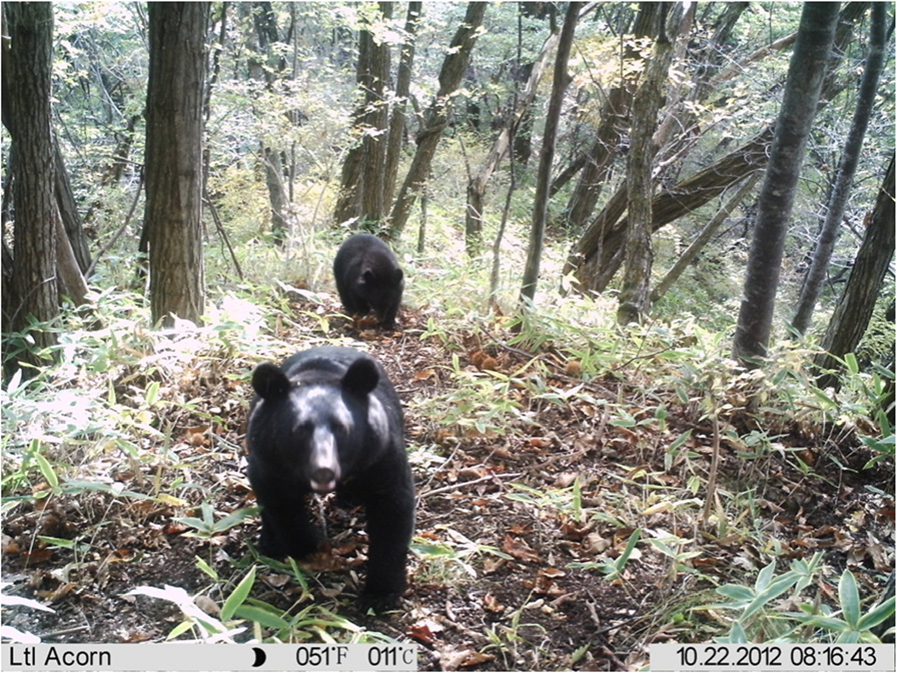
**Japanese black bears (*Ursus thibetanus japonicus*).**

**Figure 2:**
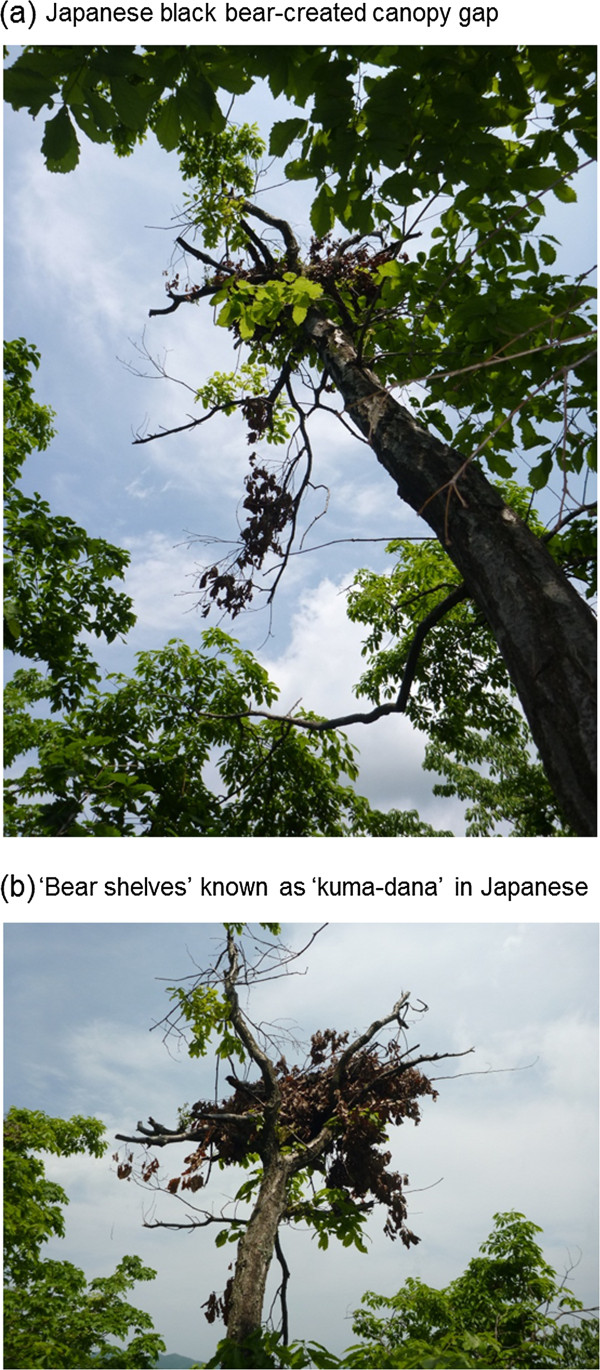
Canopy gap created by Japanese black bears and ‘bear shelf’ known as ‘kuma-dana’ in Japanese.

The Japanese black bear (*Ursus thibetanus japonicus*), a subspecies of the Asiatic black bear, is widely distributed throughout the broadleaved forests of the island of Honshu, Japan (Figure 1). These large-bodied, climbing omnivores break branches and destroy part of the crown during fruit foraging, creating small canopy gaps. No previous report has defined the canopy gaps created by black bears; here, we define these openings as gaps in the crown that are created when black bears break branches (openings without leaves or branches, as shown in Figure 2a). The bears typically disturb *Prunus* trees in early summer and *Quercus* and *Fagus* trees in autumn. At our study site in central Japan, we frequently observed gaps in the crown that were formed by foraging bears. These gaps were roughly comparable in size to tree-fall gaps formed by the falling of a single tree. Thus, similar to tree-fall gaps, Japanese black bears and the gaps they create may have the potential to alter the abiotic and biotic environment of a forest, particularly light conditions in the high and middle forest canopy. In fact, our field observations suggest that small canopy gaps created by black bears improve light conditions, which in turn facilitates fruiting by adult fleshy fruited plants located beneath the gaps (personal observations). Accordingly, the Japanese black bear may function as an ‘ecosystem engineer’ [9], altering the abiotic environment and thereby affecting surrounding biota of the forest ecosystem. However, no previous study has examined the role of canopy disturbances created by black bears in relation to light conditions and habitats of plants and/or animals. Moreover, this hypothesis that canopy disturbance by bears improve light conditions and habitats of organisms has not been thoroughly examined for the eight bear species that inhabit the world, which include polar bears (*Ursus maritimus*), giant pandas (*Ailuropoda melanoleuca*), brown bears (*Ursus arctos*), American black bears (*Ursus americanus*), Asiatic black bears (*Ursus thibetanus*), spectacled bears (*Tremarctos ornatus*), sun bears (*Helarctos malayanus*), and sloth bears (*Melursus ursinus*).

As a first step to testing this hypothesis, we collected field data to physically characterize black bear-created canopy gaps and compared these to tree-fall gaps. In general, the structure and other characteristics of tree-fall gaps have been examined in terms of size, shape, height, distribution, age of gaps, canopy openness, and light abundance [10]. Gap size is a particularly important element because it can strongly influence vegetation growth and nutrient cycling [11,12]. Canopy gaps can vary greatly in size according to the amount of material removed (e.g. ranging from single falling branches to multiple trees; [13]). Accordingly, the sizes of tree-fall gaps are typically measured using one of the following three methods: (і) assuming a uniform, elliptical shape and measuring only the major and minor axes of the gap [14]; (іі) assuming an irregular shape and measuring the distances from the center to many equally spaced points around the periphery [15]; and (ііі) assuming an irregular shape and calculating the open area using two photographs or two hemispherical photographs at different heights at the same position beneath the gap [16,17]. However, no method has been established to measure the size of small gaps caused by branch falls, as in black bear-created canopy gaps.

The purpose of this study was to characterize the physical formation of small canopy gaps created by Japanese black bears while climbing trees to reach fruit. We examined several parameters, including the tree species affected, the horizontal and vertical distribution of gaps, and the size of branches broken. We also estimated the size of small canopy gaps using two methods, which were newly devised in this study. In the first method, we took one hemispherical photograph at a position just beneath the canopy layer. In the second, we used data on the size of branches dropped to reconstruct the amount of leaf-shaded area removed from the crown. The usefulness and validity of these methods are compared in the Discussion. Furthermore, we discuss specific differences in the structure of common tree-fall gaps *versus* those created by black bears.

## Methods

### Study site

Our study site was located in a broadleaved deciduous forest in Nagakura-yama National Park, near Mt. Asama (2568 m), Nagano Prefecture, central Japan (36°22–23′ N, 138°35–38′E; 1060–1200 m a.s.l.). The annual mean temperature of this region is 8.0°C (the maximum and minimum temperature is −16.1 and 30.8°C, respectively) and the annual rainfall is 1231.9 mm, with an average maximum snow depth in winter of approximately 33.5 cm. The forest is dominated by Japanese chestnut (*Castanea crenata*), Mongolian oak (*Quercus crispula*), and Japanese white oak (*Quercus serrata*), which accounted for 28.4, 19.4, and 13.5% of the total basal area of the canopy trees (> 15 cm diameter at breast height, dbh), respectively. The other canopy tree species consisted of fleshy fruited species such as *Swida controversa* (4.5%), *Kalopanax pictus* (2.6%), *Prunus jamasakura* (1.0%), *Padus grayana* (0.8%), *Prunus sargentii* (0.7%), *Magnolia obovata* (0.7%), *Ilex macropoda* (0.6%), *Eleutherococcus sciadophylloides* (0.6%), *Prunus maximowiczii Rupr*. (0.4%), and *Prunus buergeriana* (0.1%), and wind-dispersed species such as *Ulmus davidiana* var. japonica (5.1%), *Pinus densiflora* (5.1%), *Acer mono* (3.5%), *Alnus hirsuta* (3.3%), *Acer palmatum* var. *amoenum* (2.4%), and *Acer sieboldianum* (1.3%). In the study region, Japanese black bears are not considered threatened and the study area included the core ranges of several populations in the Honshu region.

### Study plot

Plots in this study consisted of five large plots of irregular shape and 18 permanent plots of 20 × 50 m. The five large irregular plots were established in rectangular forest area of approximately 112 km^2^ between November 2006 and May 2007. The areas of the five large plots were approximately 2, 2.5, 5, 6, and 10 ha (total = 25.5 ha). The shape of each large plot was decided in order to be composed of a ridge, a slope, and a valley of the same area. Regarding the permanent plots, two, three, four, four, and five plots (total = 18 plots, 1.8 ha) of 20 × 50 m were established on each ridge in the five large plots (2, 2.5, 5, 6, and 10 ha, respectively) between April and August 2007.

### Spatial distribution of gaps

Species and topographic position of trees with black bear-created canopy gaps were recorded for all study plots between October 2007 and March 2011. Heights of the recorded trees and canopy gaps, sizes of the canopy gaps (assumed to be elliptical in shape), and GBHs (girth at breast height (1.3 m)) were measured using a SUUNTO PM5/260PC clinometer and tape measure. Gap height was determined as the position above the forest floor at which branches broken by black bears or ‘bear shelves’ were observed (known as ‘kuma-dana’ in Japanese, Figure 2b; [18]. In the 18 permanent plots, tree species and the coordinates (x, y) of all trees (> 15 cm dbh) present were recorded during the same period.

### Estimation of gap size

**Figure 3:**
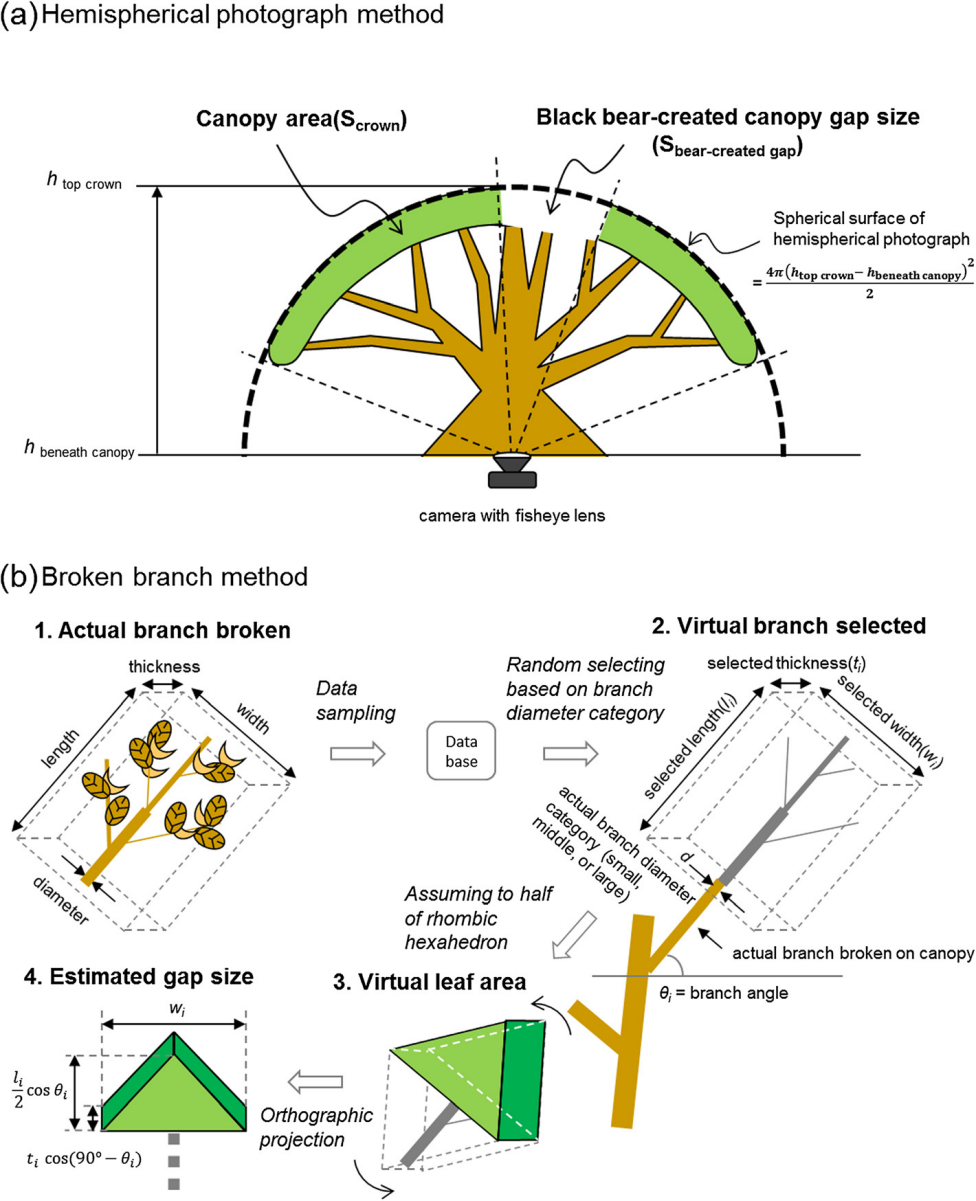
Estimation of Japanese black bear-created canopy gap size using the hemispherical photograph method and the broken branch method.

The size of black bear-created canopy gaps was estimated using two methods. The first method estimated gap size using hemispherical photographs and Gap Light Analyzer (v2.0) software ([19]; Figure 3a). Hemispherical photographs were taken at positions just beneath the crown centers of 17 Mongolian oaks (*Q. crispula*) in the 5- and 6-ha permanent plots during a foliage-on period (July–August 2007). The photographs were taken at the heights of positions beneath the canopy layer that were not covered by leaves in sub-canopy layers (average height ± SD = 9.4 ± 1.4 m). Because Mongolian oaks were dominant and were frequently disturbed by bears in the study area, we treated this species as a tree species with typical characteristics of bear-created canopy gaps. A model of the estimation method for gap size was created using data from hemispherical photographs of this tree species. We also assumed that differences in tree species had little influence on the accuracy of the model. The reason for this assumption is that, even if the tree species was not a Mongolian oak, the portions of small gaps (white area) and the crown (back area), as physical constructs, were ‘systematically’ distinguished from a photograph using Gap Light Analyzer (v2.0) software. A Nikon Coolpix 5400 digital camera with a Nikkor 7-mm FC-E9 fisheye lens and Nikon UR-E10 fisheye converter adapter was used. All measurements were taken when the sky was nearly overcast or at 1 h after dawn or 1 h before dusk.

The size of each black bear-created canopy gap was calculated using the following function:

$$S_{\mathrm{bear}-\mathrm{created}\;\mathrm{gap}}=S_{\mathrm{crown}}\times Sp_{\%\;\mathrm{bear}-\mathrm{created}\;\mathrm{gap}/\mathrm{crown}}$$

(1) $$\begin{array}{ll} = & \left\{Sp_{\%\;\mathrm{crown}}\times \frac{4\pi {\left(h_{\mathrm{top}\;\mathrm{crown}}-h_{\mathrm{beneath}\;\mathrm{canopy}}\right)}^{2}}{2}\right\}\\ & \times Sp_{\%\;\mathrm{bear}-\mathrm{created}\;\mathrm{gap}/\mathrm{crown}} \end{array}$$

where S_bear-created gap_ (m^2^) is the black bear-created canopy gap size, S_crown_ (m^2^) is the crown size, Sp_% bear-created gap/crown_ (%) is the percent of bear-created canopy gap area in relation to the total area of the crown, Sp_% crown_ (%) is the percent of the crown area in relation to the total area of a hemispherical photograph, *h*_top crown_ (m) is tree height, and *h*_beneath canopy_ (m) is the height of a position immediately below the crown of a study tree.

The second method of gap size estimation used data from branches that had been broken and dropped on the ground (Figure 3b). The length, width, and thickness of dropped branches with leaves were measured in the five large plots between October 2007 and March 2011 (Figure 3b-1). Fallen branches were categorized according to diameter (small, *d* ≤ 1.5 cm; medium, 1.5 <*d* ≤ 3 cm; and large, *d* > 3 cm). In addition, we measured the angles at which the branches had broken off at knot points (*θ* from horizontal = 0°, 15°, 30°, 45°, 60°, 75°, and 90°) during defoliation periods (November–April) in the study period. The angles of branches that were repeatedly broken in a crown were also recorded. Size data from actual branches were sampled randomly to set dimensions for a “virtual branch”, which was assumed to be a rhombic hexahedron with a densely foliated upper half that would block out sunlight (Figure 3b-2). This virtual branch was then oriented in three-dimensional space according to the branch angle data collected for each study tree/gap, and the virtual leaf area occluding sunlight was assumed to be equal to the gap existing before the branch had been broken (Figure 3b-3).

**Figure 4:**
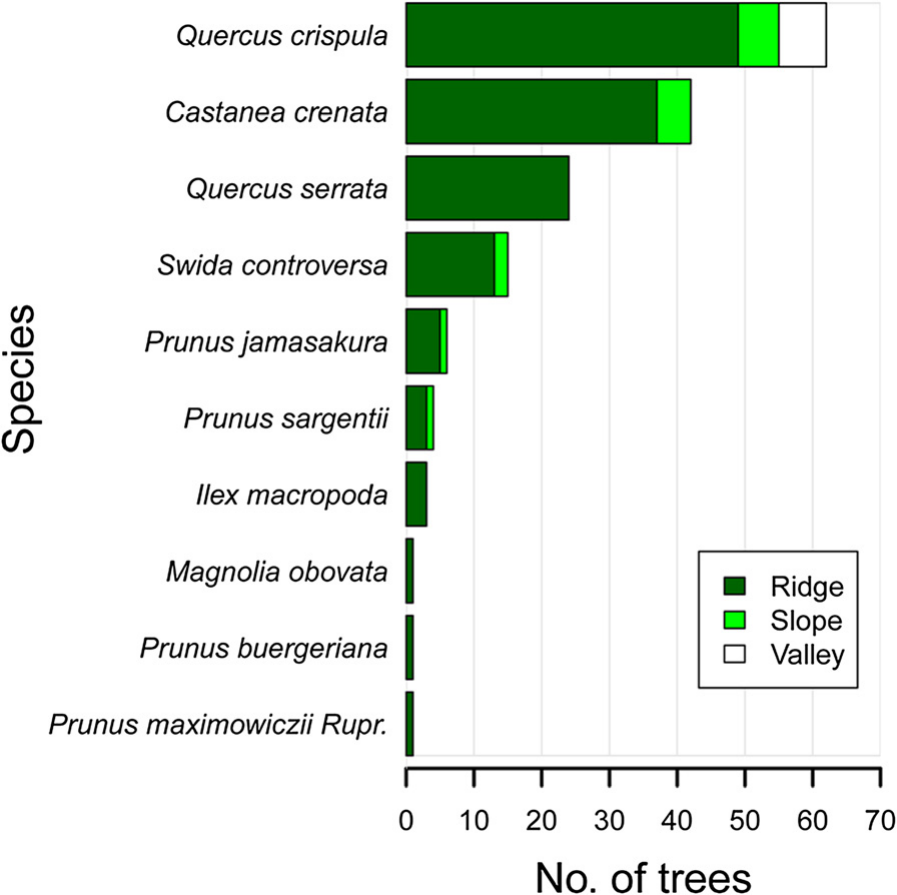
Topographic positions (ridge, slope, or valley) of trees with canopy gaps created by Japanese black bears.

The total size of black bear-created canopy gaps for each study tree was calculated using the following function:

(2) $$S_{\mathrm{bear}-\mathrm{created}\;\mathrm{gap}}=\sum_{i=1}^{n}\left\{\left(\frac{l_{i}\cos\theta_{i}\times w_{i}}{4}\right)+2\left[\frac{w_{i}}{2}\times t_{i}\cos\left(90{}^{\circ}-\theta_{i}\right)\right]\right\}$$

where S_bear-created gap_ (m^2^) is the black bear-created canopy gap size in the study tree, *l*_*i*_, *w*_*i*_, and *t*_*i*_ (m) are the length, width, and thickness of a broken branch, respectively, *θ*_*i*_ is the branch angle, and n is the number of broken branches per tree (Figure 3b-2). The first and second terms of the function show the surface area derived from length and width, and that from width and thickness of a broken branch, respectively (Figure 3b-4). In cases in which the broken branches were not found on the study plot, pooled branch size data from all other tree species were used to estimate canopy gap size.

Paired data points (estimated gap sizes) derived from the two methods were used to derive the following linear function:

(3) Shemisphericalphotographmethod=αSbrokenbranchmethod+b

where S_hemispherical photograph method_ is the canopy gap size determined by function (1), S_broken branch method_ is the canopy gap size determined by function (2), and *a* and *b* are constants. In this study, we assumed that the gap size that was calculated by the hemispherical photograph method was close to the actual value. Because estimation of the gap size by the hemispherical photograph method needs enormous labor of tree climbing, we adopted the broken branch method which is an expedient. We then attempted to derive an equation to calculate gap size using only data from broken branches. Thus, gap size within trees affected by black bear foraging activity (i.e. S_hemispherical photograph method_) was determined by substituting a value for canopy gap size (i.e. S_broken branch method_) using function (2) for function (3).

### Statistical analyses

All analyses were performed using pooled data on black bear-created canopy gaps, including gaps that were repeatedly created on the same tree, during a 5-year study period. Morisita’s index (Iδ) [20] was used to analyze the spatial distributions of both trees with or trees with and without canopy gaps. Iδ > 1, =1, and < 1 indicates a clumped, random, and uniform distribution, respectively. One-way analysis of variance (ANOVA) was used to compare the height of black bear-created canopy gaps above the forest floor, the relationship between the height of the gap and tree height (%), the size of branches dropped by black bears, the size of black bear-created canopy gaps, and the relationship between black bear-created gap size and total crown size (%). Differences in these factors across tree species were also examined using Bonferroni’s multiple comparison test. All analyses were conducted using Statistica for Windows Release 10.0 [21].

### Ethical consideration

All work was carried out within the guidelines the Mammal Society of Japan and of the Wildlife Research Center of Kyoto University.

## Results

### Horizontal distribution of gaps

In total, 159 trees with Japanese black bear-created gaps were documented throughout the entire study area over 5 years. These trees comprised 10 species, including three acorn-producing species and seven fleshy fruited species. In terms of topography, 137, 15, and 7 trees occurred on ridges, slopes, and valleys, respectively. Ridges were significantly dominant over slopes and valleys in terms of total bear-created canopy gaps (chi-square, *p* < 0.001; Figure 4).

In total, the study included 18 study plots on ridges, which contained 118 trees of eight species, including three acorn-producing species and five fleshy fruited species. Approximately 25.5% of trees on ridges were subjected to canopy disturbance by black bears over the 5-year study period and small canopy gaps were created. When analyzed according to species, the percentage of trees with canopy gaps was ordered as follows: 36.6% in *Q. crispula* (n = 52), 33.3% in *I. macropoda* (n = 2), 25.0% in *Q. serrata* (n = 18), 22.2% in *C. crenata* (n = 38), 17.6% in *P. jamasakura* (n = 3), 4.4% in *S. controversa* (n = 2), 4.4% in *P. sargentii* (n = 2), and 2.2% in *P. buergeriana* (n = 1). However, the gap formation rates in the 18 study plots on ridges did not differ significantly among tree species (ANOVA, *p* = 0.091).

**Figure 5:**
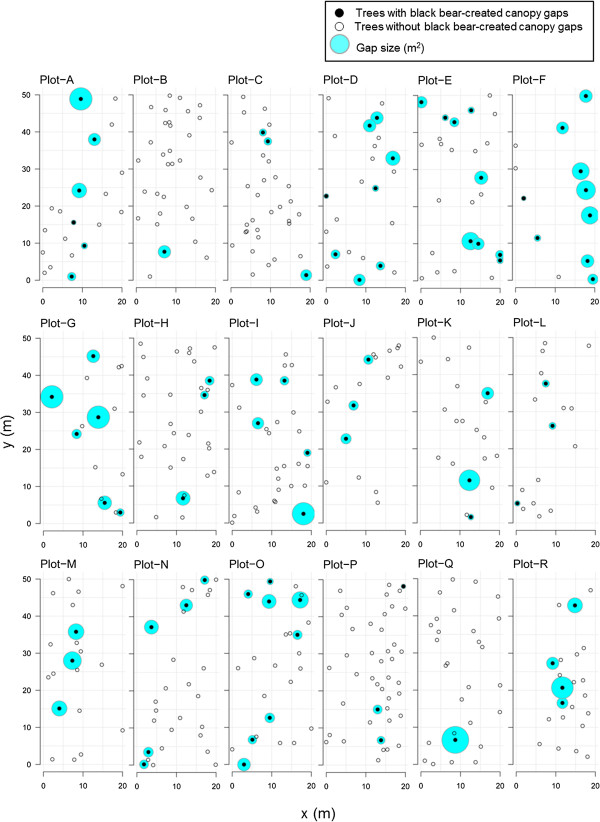
**Horizontal distribution of eight tree species (> 15 cm dbh) with (solid circles) or without (open circles) black bear-created canopy gaps.** Relative gap size is shown (open circles with gray). Affected tree species included *Quercus crispula*, *Castanea crenata*, *Quercus serrata*, *Prunus jamasakura*, *Swida controversa*, *Ilex macropoda*, *Prunus sargentii*, and *Prunus buergeriana*.

**Figure 6:**
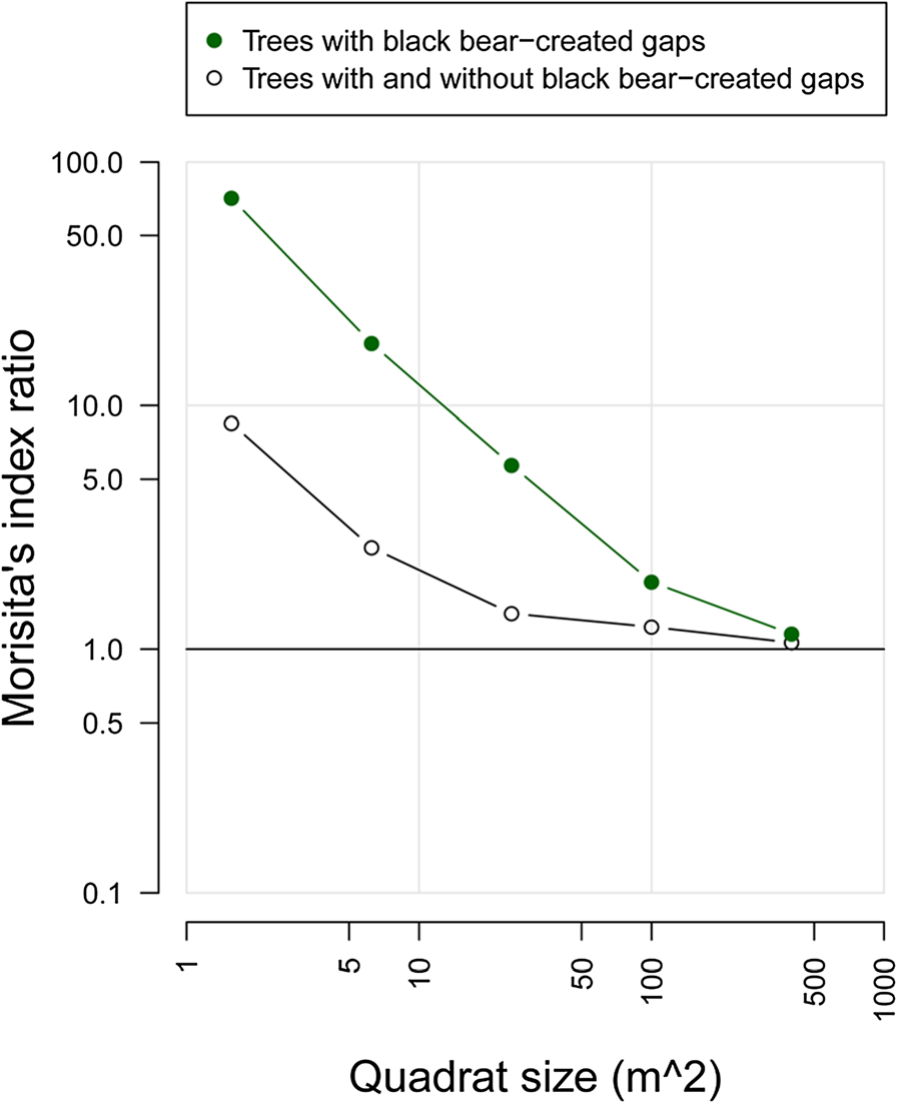
**Morisita’s index ratio (*I***_***x***_**/*I***_***x+1***_**) to quadrat size of *I***_***x***_** for horizontal distribution of trees (dbh > 15 cm) with (solid circles) or without (open circles) black bear-created canopy gaps.**

Figure 5 shows the spatial distribution of all trees > 15 cm dbh with or without small canopy gaps created by black bears over a five-year period. Across all 18 study plots, the percentage of trees showing canopy gaps ranged from 5.7% (n = 1) in Plot-Q to 28.5% (n = 9) in Plot-F. To analyze this spatial distribution, we calculated Morisita’s index (*I*_*δ*_) for both trees with or trees with and without canopy gaps and found that values were highest for quadrats of 1.25 × 1.25 m and then decreased steadily with larger quadrat size, reaching the minimum at a quadrat size of 20 × 20 m (Figure 6). In addition, the *I*_*δ*_ of trees showing black bear-created canopy gaps was larger than that of trees with and without gaps at any quadrat size (chi-square, *p* < 0.001; Figure 6). Therefore, although the initial distribution (with and without black bear-created canopy gaps) was more random, the distribution of trees with black bear-created canopy gaps was highly clumped, particularly at smaller scales.

### Vertical distribution of gaps

**Figure 7:**
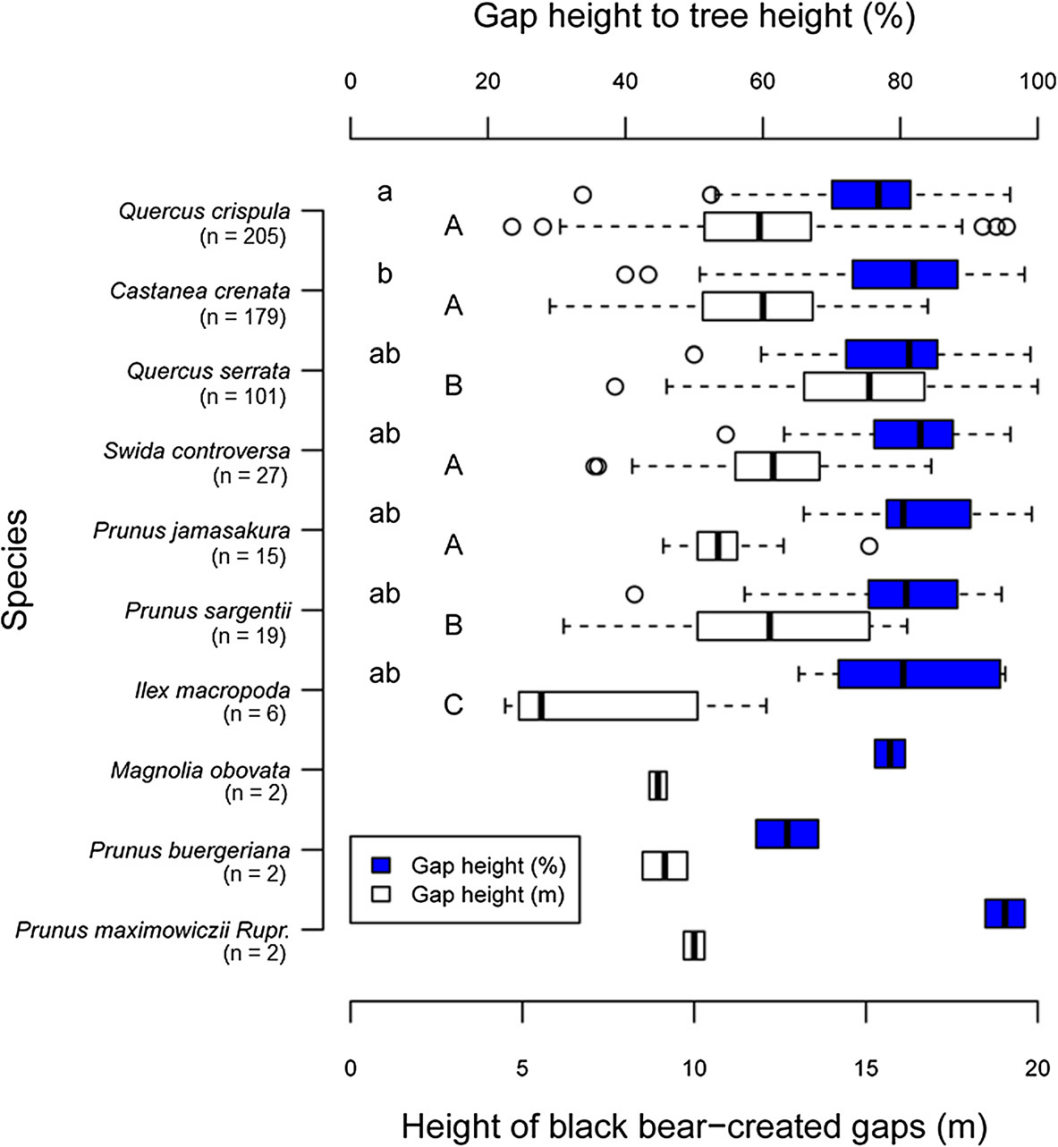
**Absolute height of Japanese black bear-created canopy gaps above the forest floor and gap height in relation to tree height (%).** Different letters show significant differences among tree species with bear-created canopy gaps (*p* < 0.05, Bonferroni’s multiple comparison test).

The absolute height of black bear-created canopy gaps and the height of these gaps relative to tree height (%) for a given tree species ranged from 4.5 m in *I. macropoda* to 22.0 m in *Q. serrata* (average = 12.4 m, SD = 2.7, n = 558) and from 33.8% in *Q. crispula* to 99.1% in *P. jamasakura* (average = 78.4%, SD = 10.1, n = 558), respectively. Therefore, although black bear-created canopy gaps were distributed throughout much of the vertical space of the crown, they tended to occur more often in upper forest layers, with heights approximately 80% above the forest floor in relation to local forest height. Gap height in relation to tree height was significantly higher in *C. crenata* than in *Q. crispula*, whereas no significant difference was observed among the other five tree species (Figure 7). The absolute height of black bear-created canopy gaps above the forest floor differed significantly according to tree species (Figure 7), being significantly higher in *Q. serrata* and *P. sargentii*, and significantly lower in *I. macropoda* (ANOVA, *p* < 0.05) .

### Broken branch size

**Figure 8:**
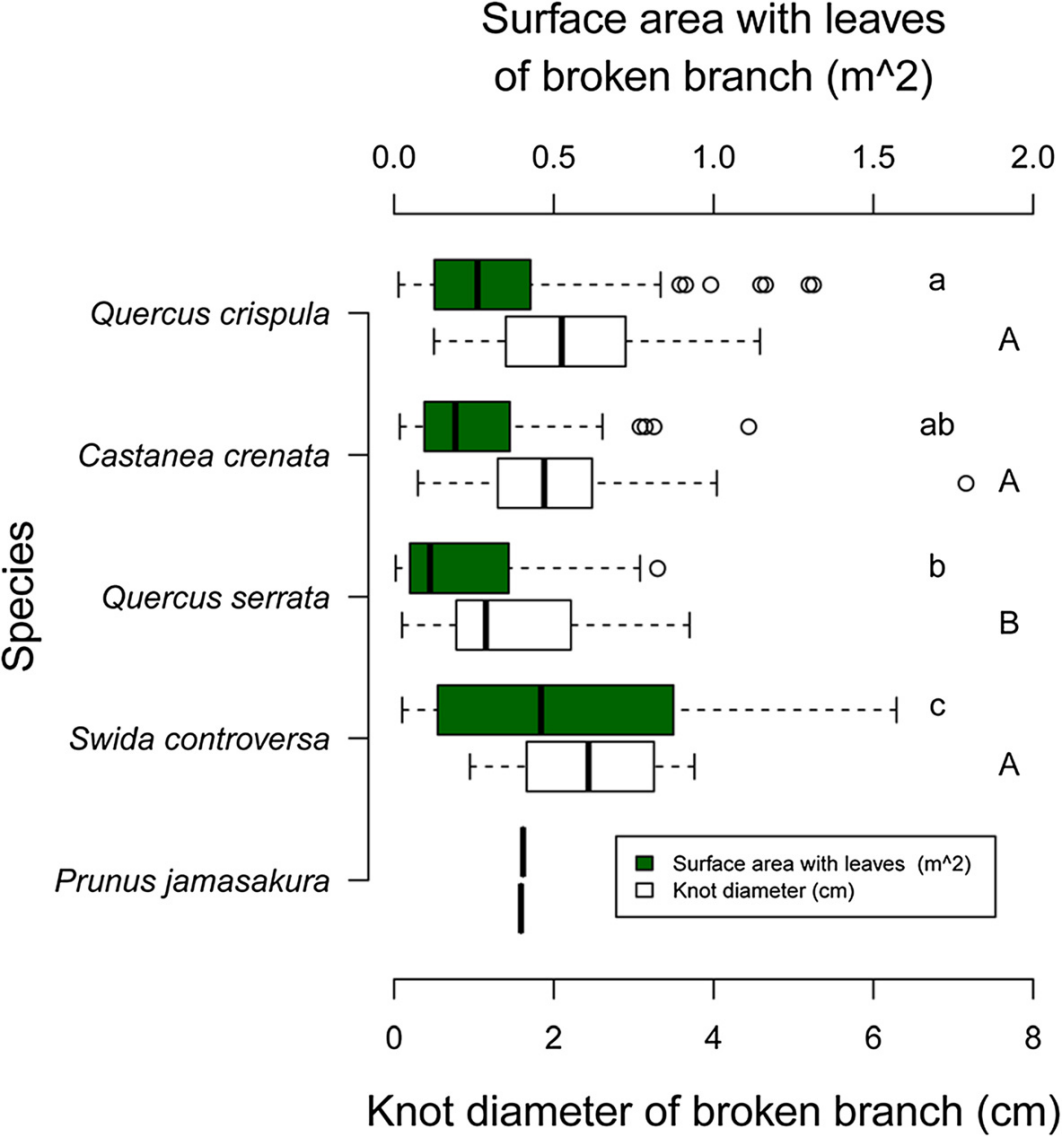
Proximate surface area with leaves, which is assumed to be the half shape of a rhombic hexahedron, and the knot diameter of branches dropped on the forest floor by foraging black bears.

The surface area of virtual branches (with leaves), which was derived from function (2) with *θ*_*i*_ equal to 0, and the knot diameter of branches that had fallen as a result of bear foraging ranged from 0.005 m^2^ in *Q. serrata* to 1.57 m^2^ in *S. controversa* (average = 0.29 m^2^, SD = 0.26, n = 325) and from 0.3 cm in *C. crenata* and *Q. serrata* to 7.2 cm in *C. crenata* (average = 2.0 cm, SD = 1.0, n = 325) (Figure 8). These values varied significantly according to tree species.

### Gap size

Estimates of black bear-created gap size obtained using the hemispherical photograph method were significantly and positively correlated with values obtained using the broken branch method (r = 0.7700, *p* = 0.0033, R^2^ = 0.5928). The relationship between values obtained through these two methods is described by the following function:

$$S_{\mathrm{hemispherical}\;\mathrm{photograph}\;\mathrm{method}}=1.1615\;S_{\mathrm{broken}\;\mathrm{branch}\;\mathrm{method}}+0.448$$

where S_hemispherical photograph method_ and S_broken branch method_ are gap size estimated using each of the methods. This formula and S_broken branch method_ were computed using data on broken branches and were used to calculate black bear-created gap sizes for all affected trees in the study area.

**Figure 9:**
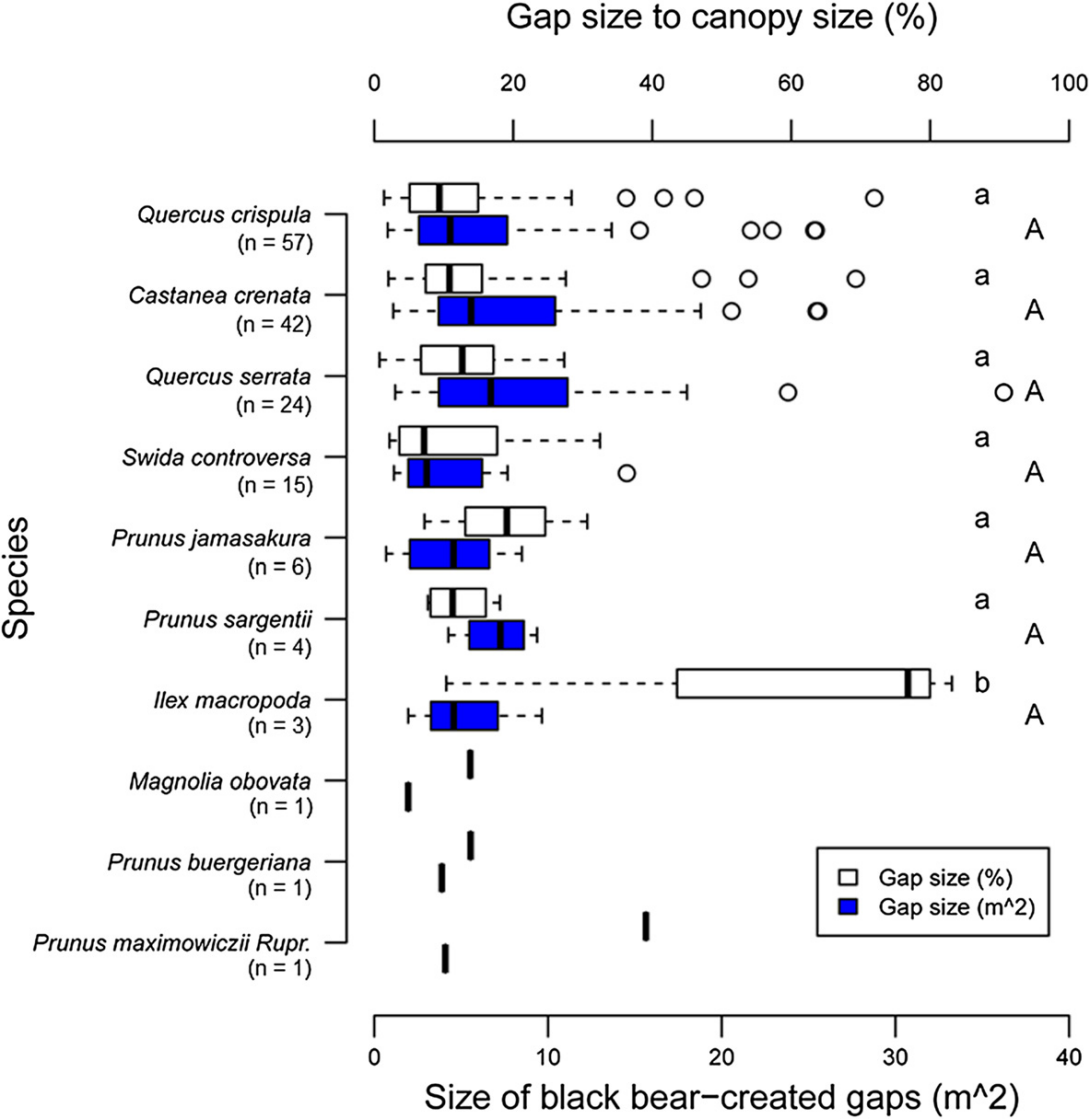
Size of Japanese black bear-created canopy gaps and gap size in relation to crown size (%).

Absolute black bear-created canopy gap size (i.e. S_hemispherical photograph method_) and gap size relative to canopy size (%) ranged from 0.7 m^2^ in *Q. crispula* to 36.2 m^2^ in *Q. serrata* (average = 6.9 m^2^, SD = 6.1, n = 154) and from 0.6% in *Q. serrata* to 98.0% in *P. jamasakura* (average = 15.0%, SD = 16.6, n = 154), respectively. These results show that black bears created small canopy gaps of diverse sizes and, furthermore, the size of black bear-created canopy gaps did not vary significantly according to tree species (Figure 9). We also noted that gap size relative to crown size tended to vary with tree species, but this effect was significant only for *I. macropoda* (Figure 9).

The relative sizes and distribution of small canopy gaps created by black bears (assumed to be circular in shape) are shown in Figure 5 for all 18 study plots at the end of the 5 years of observation. The total area of canopy gaps per study plot ranged from 129.4 m^2^ in Plot-L to 1589.6 m^2^ in Plot-F (average = 706.3 m^2^, SD = 444.3, n = 18). When converting these data to canopy gap area per hectare per year, values ranged from approximately 25.9 m^2^ ha^–1^ yr^–1^ to 317.9 m^2^ ha^–1^ yr^–1^ (average = 141.3 m^2^ ha^–1^ yr^–1^, SD = 88.9, n = 18). Moreover, because the average tree crown size (of all eight affected species) was approximately 65.1 m^2^, the number of full crowns lost (i.e. cover from an entire tree) ranged from approximately 0.4 crowns ha^–1^ yr^–1^ to 4.9 crowns ha^–1^ yr^–1^ (average = 2.2 crowns ha^–1^ yr^–1^, SD = 1.4, n = 18).

## Discussion

Our results allow for a detailed description and improved understanding of the physical characteristics and spatial distribution of canopy gaps created by Japanese black bears during foraging. The disturbance regime showed strong spatial patterning related to topography; specifically, ridges showed the highest clumped distribution of trees with black bear-created canopy gaps, whereas slopes and valleys were less affected. This topographic positioning supports previous observations by Lima and Moura [22], who reported that ridges have many small gaps created by branch falls in rain forests. In contrast, larger tree-fall gaps tend to occur on slopes and in valleys rather than on ridges, or may be fairly evenly distributed among the three topographic positions in specific forest types, such as oak forests, *Abies-Picea* forests, and rain forests [23-25]. This is because slope failures and floods initiate tree falls on slopes and valleys. In this way, we discussed on the pattern of common branch- and tree-falls in different topographic positions by reports of different forest types from our study forest. However, this pattern would be common phenomenon observed in various forest types, because the reaction of trees to different topographic positions and disturbances by wind and water is simple physical interactions. Thus, common tree-fall gaps and bear-created canopy gaps are clearly distinct in terms of topographic position. This is likely the result of the differential effects of disturbance agents (e.g. wind, water, and animals) on slopes *versus* on ridges. In our study area, more bear-created canopy gaps may have occurred on ridges because this topography facilitates bear movement and helps them to more easily approach trees with attractive fruits. The topographic distribution of tree species may also influence the formation of bear-created canopy gaps. For example, the wind-dispersed species *Ulmus davidiana* var. japonica is dominant in valleys and would not attract black bears.

However, at the small scale, we obtained much different results for the spatial distribution of tree-fall *versus* black bear-created canopy gaps. Under uniform topography, we found that bear-created canopy gaps showed a strongly clumped distribution, whereas, in general, tree-fall gaps are randomly distributed [26,27]. This can be attributed to the foraging behavior of black bears, which tend to seek out trees with large hanging fruit crops and then persist in place until these and surrounding trees are exhausted. This behavior is advantageous given that these bears require large quantities of acorns and fruits to build sufficient energy reserves for hibernation [28]. In contrast to this clumped horizontal distribution, our results showed that bear-created canopy gaps were vertically distributed throughout the canopy layers, although some tendency toward gaps in higher layers was observed. The height of a canopy gap is important to understanding its formation. Our results showed that the small canopy gaps attributed to black bears occurred lower in the crown where wind disturbance would be unlikely to break branches.

The linear function of bear-created canopy gap size derived using the hemispherical photograph method (response variable) and the broken branch method (explanatory variable) had a slope of 1.1615. Thus, gap size determination via the broken branch method underestimated gap sizes by approximately 14% compared to the hemispherical photograph method. This underestimation is likely due to conflicts in the size and position of real branches in the crown *versus* the virtual branches we devised in our model. To create virtual branches, we assumed that *i*) the leafy part of a broken branch occupied systematically half of the flattened area of a rhombic hexahedron derived through random sampling of the data, as is shown in Figure 3b-2, and that *ii*) these leafy areas could be mathematically summed to completely fill the gap, with no open space. The former assumption is because a rhombic hexahedron is a polyhedron that most closely resembled a real branch, and also because leafy part of a broken branch concentrates in half of a rhombic hexahedron. Despite this error, our linear model showed a relatively good fit for the relationship between values obtained via the broken branch method *versus* the hemispherical photograph method (R^2^ = 0.5928). Therefore, our data suggest that the broken branch method is an effective strategy to estimate the total area of many small canopy gaps occurring in a comparatively large stand.

Japanese black bears are extremely strong and can cause highly focused damaged in the canopy. The size of broken branches differed among tree species, probably as a result of differences in the density of fruits per branch, branch strength, and ease of handling the branches. However, a wide range of canopy gap sizes was observed across tree species, indicating that tree species did not affect the size of canopy gaps caused by black bears. Although the reason for this is unclear, it implies that black bears create small canopy gaps by breaking one branch as well as larger gaps by breaking multiple branches, irrespective of tree species. However, our results predict that the total area of black bear-created canopy gaps in a stand does depend on species composition; for example, stands with an abundance of fruit-bearing trees will show a larger overall canopy loss.

In previous studies on temperate broadleaved forests dominated by Fagaceae, the size of tree-fall gaps caused by windfall of one or multiple trees ranged from 10 to >1000 m^2^[11,29-32]. In contrast, canopy gaps caused by bears are relatively small. In this study, only 19.5% of the trees affected showed black bear-created canopy gaps ≥ 10 m^2^, which is roughly the minimum size of a tree-fall gap. However, although bear-created canopy gaps are individually smaller, the mean size of canopy openings reached 141.3 m^2^ ha^–1^ yr^–1^ on ridges, which were hot spots of bear activity. This area corresponded to the crown area of 2.2 trees. In our study area, gap formation by falling of three trees was observed in permanent plots (1.8 ha) on ridges within the 5-year study period. The annual gap formation rate by tree falls was approximately 21.4 m^2^ ha^–1^ yr^–1^. Surprisingly, the annual rate of black bear-created gap formation was approximately 6.6 times that of tree-fall gap formation. Also, the rate of bear-created gap formation was demonstrably higher than that of tree-fall gap formation in several previous studies: 42.0 m^2^ ha^–1^ yr^–1^ in an old-growth deciduous forest [33], 55.6 m^2^ ha^–1^ yr^–1^ in an evergreen broadleaf forest [34], and 81.3 m^2^ ha^–1^ yr^–1^ in a conifer/broadleaved forest [35] in Japan. Therefore, canopy gap creation by black bears represents a significant disturbance that affects canopy dynamics in this region. However, it was not clear if our finding of high-density canopy gap creation represents a special case or not. There are no reports of black bear-created canopy gaps and bear shelves elsewhere in the world, and thus we were not able to compare our study area with other forests. Canopy gap creation by black bears would also vary depending on year-to-year fluctuations in seed production and black bear populations. In temperate deciduous forests, acorn-producing and fleshy fruited tree species have larger fluctuations in annual seed production at the population level [35,36]. This suggests that the density of black bear-created canopy gaps will be higher in good mast years and in regions with high densities of black bears, such as the study area. The population size in this area was approximately 3.3–3.9 individuals/km2 [37].

At present, the distribution of black bears is heterogeneous in broadleaved forests of Japan. Many parts of the main island of Honshu have high bear densities, whereas few to no bears inhabit western Honshu and the islands of Shikoku and Kyushu. Several populations in western Japan are designated as endangered in the Red Data Book of Japan [38]. These regions would accordingly have little to no black bear-driven canopy disturbance, and it is unknown how this may affect ecosystem function and biodiversity among regions with or without black bears. Much more research is required to examine the role of black bear-created canopy gaps, as well as tree-fall gaps, in forest ecosystems in terms of both local effects and regional differences.

## Conclusions

Consequently, our data demonstrate that foraging black bears play an important role in canopy disturbance. This disturbance regime was characterized by highly clumped gap formation on ridges (on the large scale), a large overall affected area, a broad vertical distribution of canopy gaps, and a wide range in gap size. Moreover, the largest of the black bear-created canopy gaps were roughly equal to general gap disturbances caused by tree falls. Being smaller and differentially distributed, bear-created canopy gaps may uniquely alter the light regime compared to tree-fall gaps, resulting in an ecological interaction between black bears and trees producing attractive fruits. By increasing available light, black bear-created canopy gaps may be an important factor in the growth, reproduction, and recruitment of plants below the canopy. Such a role would indicate that black bears are in fact ‘ecosystem engineers’ that directly or indirectly modulate the availability of resources to other species by causing physical state changes in biotic and abiotic materials [9]. However, the role of small gaps created by black bears may be different from that of common tree-fall gaps which are larger in size and have slower turnover. We were not able to perform a detailed study of the recovery of gaps because there is no established method for measuring the recovery of small-sized gaps. Actually, it was very difficult to distinguish whether a gap was made repeatedly in an old gap or if it was a new gap in the same crown. Future studies should estimate canopy turnover rates, which are affected by the balance between the recovery of gaps and repeated gap creation by black bears. This information would help to clarify the generality and the role of these small gaps in forest dynamics and tree communities.

## Competing interests

The authors declare that they have no competing interests.

## Authors’ contributions

Both authors generated the original idea, designed the research, and carried out the field work. Kazuaki T identified species, analyzed field data, and drafted the manuscript. Both authors read and approved the final manuscript.
